# A molecular target of vascular calcification in chronic kidney disease

**DOI:** 10.1172/JCI156257

**Published:** 2022-01-04

**Authors:** Mohamed G. Atta

**Affiliations:** Johns Hopkins University School of Medicine, Baltimore, Maryland, USA.

## Abstract

Vascular calcification (VC) causes cardiovascular morbidity and mortality in patients with chronic kidney disease (CKD), particularly those with end-stage kidney disease (ESKD) on maintenance dialysis treatment. Although many mechanisms have been proposed, their detailed effects remain incompletely understood. In this issue of the *JCI*, Li et al. examined the molecular mechanism of the protective role of SIRT6 in VC in patients with CKD. Using in vitro and animal models of CKD, the authors demonstrated that SIRT6 prevents VC by suppressing the osteogenic transdifferentiation of vascular smooth muscle cells (VSMCs). Mechanistically, SIRT6 bound and deacetylated the runt-related transcription factor 2 (Runx2), a key transcription factor for osteogenic differentiation, promoting its nuclear export for proteasome degradation. These studies provide a pathway in the pathogenesis of VC and justify investigating SIRT6 as a potential target in CKD.

## Evolution of vascular calcification

**Figure 1:**
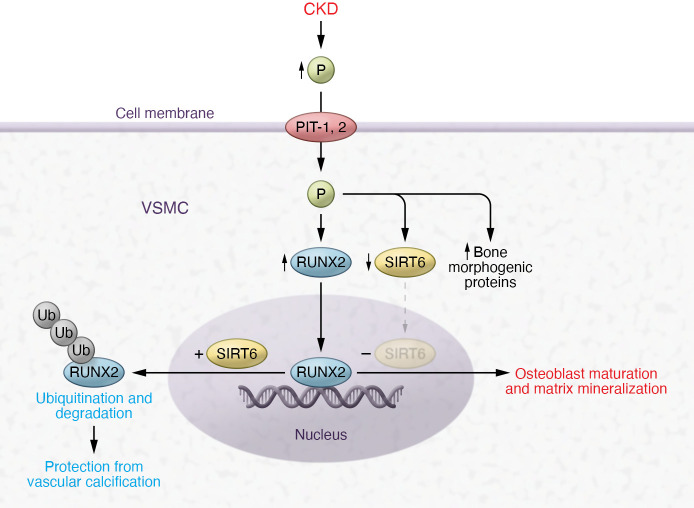
Mechanism for osteogenic transdifferentiation of VSMCs in the pathogenesis of VC. VSMCs uptake phosphorus via the sodium-dependent phosphate cotransporters PiT-1 and PiT-2 in response to increases in extracellular phosphorus of any cause, most commonly in patients with CKD. This uptake leads to downregulation of SIRT6 expression, upregulation of bone morphogenic genes, and an increase in the expression of the transcription factor Runx2, the master regulator of bone formation. In the absence of SIRT6, Runx2 signals osteoblast maturation and matrix mineralization. In contrast, overexpression of SIRT6 marks Runx2 protein for ubiquitination and degradation, preventing VSMC calcification. The study by Li et al. (16) showed that SIRT6 upregulation in an environment of increased extracellular phosphorus leads to SIRT6 binding and deacetylation of Runx2 protein in VSMCs, promoting its nuclear export for proteasome degradation and thus preventing its downstream signal for osteogenic transdifferentiation.

Vascular calcification (VC) involves the deposition of calcium phosphate mineral along with the local expression and deposition of bone-associated, mineralization-regulating proteins at two distinct sites of the blood vessel: the intima and the media (1). In contrast to the atherosclerotic intimal calcification, medial calcification of the VSMCs, commonly seen in patients with advanced chronic kidney disease (CKD), occurs in the absence of lipids or inflammatory cells. An increase in extracellular calcium and phosphorus from any cause favors VC. In vitro experiments and animal models demonstrate that inorganic phosphorus enters the VSMCs via the sodium-dependent phosphate cotransporters PiT-1 and PiT-2 (Figure 1), increasing mineral deposition in a time- and concentration-dependent manner (2).

A high-phosphate environment is common in the setting of CKD, with patients possessing a net positive phosphorus balance. As kidney function declines, maintenance of normal serum phosphate levels depends on two phosphaturic hormones: fibroblast growth factor 23 (FGF23), secreted by bone osteocytes, and parathyroid hormones (PTH), released from the parathyroid gland. PTH binds to the PTH type 1 receptor on the basolateral membrane of the proximal tubular cells, inducing the retrieval of sodium phosphate transporter 2a (NPT2a) from the brush border. Similarly, FGF23 and its cofactor klotho bind to the klotho-FGFR1c complex on the basolateral membrane of the proximal tubular cells, which decreases the expression of both sodium phosphate cotransporters NPT2a and NPT2c. The central role of the FGF23-klotho complex in phosphate regulation is shown by the development of vascular and ectopic calcifications and the short lifespan in klotho-deficient mice or double knockout of both klotho and FGF23 in mice on a high-phosphate diet (3, 4). Adaptive mechanisms of phosphate regulation are lost with the progression of CKD and particularly in end-stage kidney disease (ESKD) leading to increased extracellular phosphorus and a rise in VSMC uptake of phosphorus.

VC is actively inhibited in VSMCs (5, 6) but with dysregulated mineral metabolism in CKD, calcification begins in the tunica media, ultimately leading to concentric thickening of the vessel wall (7). VSMCs derived from the calcification lesions exhibit osteoblastic properties, with VSMCs expressing markers of osteogenesis. Among the upregulated bone morphogenic genes expressed in VSMCs treated with high phosphorus are osteocalcin, the bone morphogenetic proteins (BMPs), and Runx2, previously called Osf2 or Cbfa-1 (2, 8). BMP proteins extracted from bone are capable of inducing cartilage and bone formation when injected subcutaneously, intramuscularly, or periosteally into adult rats or other species (9, 10). Recently, activation of BMP2 via alkB homolog 1 overexpression was shown to increase the progression and severity of VC by increasing the expression of Runx2 (11). The master regulator of bone formation is the transcription factor Runx2, and haploinsufficiency of Runx2 causes the hereditary bone disease cleidocranial dysplasia (12, 13). Moreover, targeted disruption of Runx2 results in a complete lack of bone formation due to maturational arrest of osteoblasts (14). The activity of Runx2 protein is regulated by various posttranslational modifications such as phosphorylation, methylation, acetylation, or ubiquitination (15). This regulation suggests that suppression of any of the Runx2 posttranslational modifications may aid in the prevention of VC.

## Ubiquitination of the Runx2 protein

In this issue of the *JCI*, Li et al. (16) elegantly unraveled a mechanism of repressing Runx2 signaling. The authors showed that SIRT6 played an essential role in the posttranslational modification of Runx2 suppressing osteogenic transdifferentiation of VSMCs and subsequently inhibited VC in CKD. The expression of SIRT6 was decreased in peripheral blood mononuclear cells and calcified arteries from patients with CKD and CKD animal models (both adenine-induced CKD and 5/6 nephrectomy CKD mouse models). Reversal of VC was shown in SIRT6-transgenic (SIRT6-Tg) mice overexpressing SIRT6. In contrast, silencing SIRT6 in vitro with small interfering RNA (siRNA) or the specific SIRT6 inhibitor OSS-128167 resulted in VC. Similarly, a mouse model with specific adeno-associated virus–mediated (AAV-mediated) SIRT6 knockdown exhibited more severe VC than mice in the control group. Finally, the role of the SIRT6-Runx2 interaction in VC was validated in series of in vivo and in vitro experiments. Specifically, the authors demonstrated that Runx2 protein expression was decreased in SIRT6-Tg mice overexpressing SIRT6, and that enhancing Runx2 expression via plasmid reversed the protective effect of SIRT6 in vitro. The authors showed that decreased protein expression of Runx2 occurs via posttranslational regulation (decreased Runx2 acetylation marked the protein for ubiquitination and degradation) and not at the transcription level. Further, the physical interaction of SIRT6 with Runx2 was demonstrated by coimmunoprecipitation assays.

## A red berry a day keeps VC away?

Sirtuins are NAD^+^-dependent enzymes and among the sirtuins family 1 to 7, SIRT6 has been implicated in protecting against diverse conditions such as aging, age-related diseases, and metabolic disease (17–19). Flavonoids and specifically anthocyanidins modulate SIRT6 activity (20). Cyanidin (present to a high degree in red berries) is the most potent compound that substantially increases the deacetylation activity of SIRT6. The study by Li et al. (16) showed that SIRT6 enhances RUNX2 ubiquitination and degradation, inhibiting VC (Figure 1). However, the study also reflects on the complexity of osteogenic transdifferentiation of VSMCs and the multitude of signaling pathways involved in this process. Many therapeutic attempts to counter the process of VC focused on the upstream pathway signals by targeting phosphorus and mineral metabolism dysregulation as an initiator of vascular toxicity. None has unequivocally shown promising results in reversing VC or reducing cardiovascular events in patients with ESKD (21–24). A large clinical trial (PHOSPHATE) is currently underway to test the impact of intensive versus liberal phosphate target on composite cardiovascular outcome in ESKD on dialysis (NCT03573089). SNF472, the hexasodium salt of phytate, is being developed as an intravenously administered form of phytate to prevent phosphate and calcium aggregate into hydroxyapatite crystals into VSMCs, and has been shown to reduce the progression of coronary artery calcium and aortic valve calcification in patients with ESKD receiving hemodialysis (25). One may argue that interventions in ESKD are too late to reverse VC and that interventions in earlier stages of CKD are difficult to implement since these patients have normal-looking mineral homeostasis. Interventions at the downstream signals of the VC processes have inherent complications. There are many osteogenic signaling pathways that ultimately result in Runx2 activation. Selection of the appropriate pathway, timing of intervention, and avoiding off target effects are challenging tasks. Would suppressing Runx2 result in osteoporosis? Would SIRT6 upregulation to enhance Runx2 ubiquitination and degradation serve better than directly inhibiting Runx2 expression in reversing VC? Would eating a red berry a day keep VC away? Despite the many questions, the study by Li et al. (16) provides a pathway in the pathogenesis of VC that warrants further testing. The study was well executed with an impactful finding and lays the foundation for SIRT6 as a potential druggable target.

## Version 1 01/04/2022

Electronic publication

## References

[1] Proudfoot D, Shanahan CM (2001). Biology of calcification in vascular cells: intima versus media. Herz.

[2] Jono S (2000). Phosphate regulation of vascular smooth muscle cell calcification. Circ Res.

[3] Kuro-o M (1997). Mutation of the mouse klotho gene leads to a syndrome resembling ageing. Nature.

[4] Ohnishi M, Razzaque MS (2010). Dietary and genetic evidence for phosphate toxicity accelerating mammalian aging. FASEB J.

[5] Bucay N (1998). Osteoprotegerin-deficient mice develop early onset osteoporosis and arterial calcification. Genes Dev.

[6] Luo G (1997). Spontaneous calcification of arteries and cartilage in mice lacking matrix GLA protein. Nature.

[7] Shanahan CM (1999). Medial localization of mineralization-regulating proteins in association with Mönckeberg’s sclerosis: evidence for smooth muscle cell-mediated vascular calcification. Circulation.

[8] Li X (2008). BMP-2 promotes phosphate uptake, phenotypic modulation, and calcification of human vascular smooth muscle cells. Atherosclerosis.

[9] Reddi AH (1997). Bone morphogenetic proteins: an unconventional approach to isolation of first mammalian morphogens. Cytokine Growth Factor Rev.

[10] Sampath TK, Reddi AH (1981). Dissociative extraction and reconstitution of extracellular matrix components involved in local bone differentiation. Proc Natl Acad Sci U S A.

[11] Ouyang L (2021). ALKBH1-demethylated DNA N6-methyladenine modification triggers vascular calcification via osteogenic reprogramming in chronic kidney disease. J Clin Invest.

[12] Mundlos S (1999). Cleidocranial dysplasia: clinical and molecular genetics. J Med Genet.

[13] Wysokinski D (2015). RUNX2: a master bone growth regulator that may be involved in the DNA damage response. DNA Cell Biol.

[14] Komori T (1997). Targeted disruption of Cbfa1 results in a complete lack of bone formation owing to maturational arrest of osteoblasts. Cell.

[15] Kim WJ (2020). RUNX2-modifying enzymes: therapeutic targets for bone diseases. Exp Mol Med.

[16] Li W (2022). SIRT6 protects vascular smooth muscle cells from osteogenic transdifferentiation via Runx2 in chronic kidney disease. J Clin Invest.

[17] Kanfi Y (2012). The sirtuin SIRT6 regulates lifespan in male mice. Nature.

[18] Mostoslavsky R (2006). Genomic instability and aging-like phenotype in the absence of mammalian SIRT6. Cell.

[19] Chalkiadaki A, Guarente L (2015). The multifaceted functions of sirtuins in cancer. Nat Rev Cancer.

[20] Rahnasto-Rilla M (2018). Natural polyphenols as sirtuin 6 modulators. Sci Rep.

[21] Habbous S (2017). The efficacy and safety of sevelamer and lanthanum versus calcium-containing and iron-based binders in treating hyperphosphatemia in patients with chronic kidney disease: a systematic review and meta-analysis. Nephrol Dial Transplant.

[22] Investigators ET (2012). Effect of cinacalcet on cardiovascular disease in patients undergoing dialysis. N Engl J Med.

[23] Jamal SA (2009). The effects of calcium-based versus non-calcium-based phosphate binders on mortality among patients with chronic kidney disease: a meta-analysis. Nephrol Dial Transplant.

[24] Jamal SA (2013). Effect of calcium-based versus non-calcium-based phosphate binders on mortality in patients with chronic kidney disease: an updated systematic review and meta-analysis. Lancet.

[25] Raggi P (2020). Slowing progression of cardiovascular calcification with SNF472 in patients on hemodialysis: results of a randomized phase 2b study. Circulation.

